# Leveraging machine learning to enhance appointment adherence at a novel post-discharge care transition clinic

**DOI:** 10.1093/jamiaopen/ooae086

**Published:** 2024-11-09

**Authors:** Seung-Yup Lee, Reid M Eagleson, Larry R Hearld, Madeline J Gibson, Kristine R Hearld, Allyson G Hall, Greer A Burkholder, Jacob McMahon, Shoaib Y Mahmood, Corey T Spraberry, Thalia J Baker, Alison R Garretson, Heather M Bradley, Michael J Mugavero

**Affiliations:** Department of Health Services Administration, The University of Alabama at Birmingham, Birmingham, AL 35233, United States; Center for Outcomes and Effectiveness Research and Education, The University of Alabama at Birmingham, Birmingham, AL 35233, United States; Department of Health Services Administration, The University of Alabama at Birmingham, Birmingham, AL 35233, United States; Center for Outcomes and Effectiveness Research and Education, The University of Alabama at Birmingham, Birmingham, AL 35233, United States; Department of Health Services Administration, The University of Alabama at Birmingham, Birmingham, AL 35233, United States; Department of Health Services Administration, The University of Alabama at Birmingham, Birmingham, AL 35233, United States; Department of Medicine, The University of Alabama at Birmingham, Birmingham, AL 35294, United States; College of Science and Mathematics, Auburn University, Auburn, AL 36849, United States; UAB Medicine, Birmingham, AL 35233, United States; UAB Medicine, Birmingham, AL 35233, United States; UAB Medicine, Birmingham, AL 35233, United States; UAB Medicine, Birmingham, AL 35233, United States; Cooper Green Mercy Health Services Authority, Birmingham, AL 35233, United States; Center for Outcomes and Effectiveness Research and Education, The University of Alabama at Birmingham, Birmingham, AL 35233, United States

**Keywords:** machine learning, patient compliance, health services research, appointments and schedules, electronic health records, healthcare disparities, risk factors

## Abstract

**Objective:**

This study applies predictive analytics to identify patients at risk of missing appointments at a novel post-discharge clinic (PDC) in a large academic health system. Recognizing the critical role of appointment adherence in the success of new clinical ventures, this research aims to inform future targeted interventions to increase appointment adherence.

**Materials and Methods:**

We analyzed electronic health records (EHRs) capturing a wide array of demographic, socio-economic, and clinical variables from 2168 patients with scheduled appointments at the PDC from September 2022 to August 2023. Logistic regression, decision trees, and eXtreme Gradient Boosting (XGBoost) algorithms were employed to construct predictive models for appointment adherence.

**Results:**

The XGBoost machine learning model outperformed logistic regression and decision trees with an area under the curve (AUC) of 72% vs 65% and 67%, respectively, in predicting missed appointments, despite limited availability of historical data. Key predictors included patient age, number of days between appointment scheduling and occurrence, insurance status, marital status, and mental health and cardiac disease conditions.

**Discussion:**

Findings underscore the potential of machine learning predictive analytics to significantly enhance patient engagement and operational efficiency in emerging healthcare settings. Optimizing predictive models can help balance the early identification of patients at risk of non-adherence with the efficient allocation of resources.

**Conclusion:**

The study highlights the potential value of employing machine learning techniques to inform interventions aimed at improving appointment adherence in a post-discharge transition clinic environment.

## Introduction

Hospitals are responsible for ensuring effective care transitions from acute care to outpatient settings. Inadequate follow-up, particularly for patients with ongoing medical needs and limited access to primary care, can escalate hospital readmissions and emergency department (ED) visits, thereby straining hospital operations and finances.^1–3^ Post-discharge care interventions can bridge this transition gap.^4^ Research has shown that timely ED follow-up is associated with a reduced likelihood of return to the acute care setting^5^ and is important for ensuring that patients are able to follow treatment plans and are aware of the symptoms associated with their illness.^6^

Post-discharge clinics (PDCs) are dedicated to providing follow-up care to recently discharged patients and have emerged as a key strategy for care transitions and preventable health care utilization.^7–10^ The potential for PDCs to reduce readmission rates and enhance post-discharge outcomes has garnered interest from many U.S. hospitals, particularly following the introduction of programs like the Hospital Readmission Reduction Program, where hospitals with higher-than-expected readmission rates for selected conditions receive reduced payments.^11^^,^^12^

Despite these potentially significant benefits, patient adherence to treatment plans, such as attending outpatient appointments, remains a major barrier to maximizing the effectiveness of post-discharge transitional care services.^13^^,^^14^ Novel predictive analytic techniques may improve patient prioritization and engagement by identifying patients at elevated risk of missing appointments. In the outpatient setting, various machine learning approaches, such as gradient boosting, deep learning, and resampling techniques, have been utilized to predict hospital outpatient non-attendance.^15–17^ Outpatient appointment adherence studies have also explored disease-specific predictions, where clinical implications are significant.^18–20^ However, because PDCs are conceptually new, evidenced-based protocols for supporting adherence to PDC appointments remain largely underexplored. Given the issue of high no-show rates in the PDC setting (eg, reaching 50%),^21^ integrating such nuanced analyses with electronic health record (EHR) data can help identify patients at risk of appointment non-adherence and, in turn, enable targeted interventions for those most likely to miss post-discharge care.

Our study uses predictive machine learning with EHR data from a large academic medical center in the Southeastern United States to identify patients at higher risk of appointment non-adherence at a new PDC following inpatient hospitalization or ED visit. Such efforts are especially important during the early stages of a new clinic’s implementation when organizational routines are not yet “hardwired” into social and technical norms, thus providing a unique opportunity to develop and implement evidence-based interventions to support appointment adherence. By informing targeted interventions with machine learning predictions, we anticipate enabling more efficient clinic operations and improved patient outcomes, thereby mitigating the “liability of newness” challenge that new healthcare ventures often face.^22–24^

In this analysis, we employ 3 distinct analytical models: logistic regression, decision tree, and eXtreme Gradient Boosting (XGBoost). Logistic regression, a traditional linear method, provides a foundation for understanding basic relationships within data. By contrast, decision trees can capture non-linear relationships and mimic human decision-making processes but lack flexibility. XGBoost is an advanced machine learning technique that excels in handling such complexities by detecting intricate patterns and interactions.^25–27^ This multi-method approach allows us to evaluate the merits of applying predictive analytics with different levels of computational complexity, demonstrating the potential of advanced techniques to bolster patient appointment adherence.

## Methods

### Study setting

Established in September 2022, the PDC at the University of Alabama at Birmingham (UAB) offers follow-up care to patients within 14 days of discharge from any UAB ED or Hospital. UAB Medicine is a comprehensive tertiary care academic medical center in Birmingham, AL with over 1200 inpatient beds and approximately 135 000 ED visits and 409 000 inpatient days each year, spanning 2 hospitals and 3 EDs.

Upon discharge from the ED or hospital, if the patient is not connected to a primary care clinic, the discharge team refers the patient to the PDC based on their condition. If the patient is already linked to a primary care clinic, the discharge team first contacts the primary care clinic, and a PDC visit is arranged. The goal is to ensure that the appointment is scheduled to take place within 7 to 14 days post-discharge.

The PDC was managed by a Physician/Medical Director and an Advanced Practice Practitioner, who dedicated 50% and 90% of their full-time equivalents to clinical services, respectively. The support team, including receptionists and social workers, was shared with other clinics within the same ambulatory care facility. In essence, the PDC was integrated into UAB Medicine’s care network, aligning with the broader mission of the medical center to enhance care transitions and reduce ED visits and hospitalizations. In keeping with UAB Medicine’s mission to serve underserved populations in the region, the PDC charged a minimum deposit or varying amounts up to the full cost of services, depending on the patient’s eligibility (such as charity approval), for uninsured or self-pay patients.

### Data

Our dataset included 2168 appointments from 1716 patients during the first operational year of the PDC (September 2022 to August 2023). Appointment data were anonymized for patient confidentiality and included dates and times when appointments were scheduled and set to occur, the type of appointment (following an ED visit or hospital discharge), medical history, the number of days between appointment scheduling and occurrence (lag days), and the status of the appointment (such as arrived, no show, canceled by patient, or bumped). A bumped appointment refers to one that the clinic rescheduled for operational reasons. The demographic and socio-economic data included city, state, zip code, age, gender, ethnicity, race, marital status, preferred language, religion, primary insurance provider, and specific needs (eg, wheelchair and hearing impaired). These data are vital to assessing and predicting healthcare access and utilization after discharge and to pinpointing potential disparities in care, as highlighted in previous studies.^12^^,^^28^

Appointment status (ie, appointment adherence) was the primary outcome variable. We combined the “no show” and “canceled” categories to create a “missed” appointment category in our binary classification models. “Arrived” appointments served as the negative class in our models. Thirteen appointments were “bumped” and excluded from the analysis, leaving 2155 observations. All other variables, including demographic, socio-economic, and clinical variables, were tested as predictors of appointment adherence.

While UAB Medicine, as a comprehensive tertiary care academic medical center, maintains a well-structured and reliable EHR entry system, our dataset was not entirely free of missing values. The missing value rates for race/ethnicity, primary insurance provider, marital status, preferred language, and religion were 1.9%, 1.9%, 2.4%, 2.4%, and 14.8%, respectively. These missing values were coded as “Unknown” and treated as a separate category within each categorical variable. Notably, there were no missing values for age, gender, and lag days. The “specific needs” variable had 97.6% of missing values; however, this was because these cases did not have any specific needs during their ED visit or hospitalization. Therefore, we categorized these as “Not applicable” for the specific needs variable. Lastly, binary medical history data were collected for each specific chronic disease diagnosis, with 1 indicating presence and 0 indicating absence. This format allowed us to represent the presence of multiple chronic diseases. Our dataset included a total of 33 unique medical history items.

### Predictive analysis methodologies

We utilized logistic regression, decision trees, and XGBoost methods to analyze the dataset and test their effectiveness in predicting appointment adherence. We chose these 3 methods to represent a traditional statistical inference-based model, an interpretable machine learning model, and an advanced machine learning model, respectively. The first 85% of observations were designated for model training, where the algorithms learn to recognize patterns and relationships in the data, while the remaining 15% of unseen data were reserved for testing predictive accuracy and generalizability.

Logistic regression, a traditional statistical model, is simple, easily interpretable, and a staple in biomedical research.^29^^,^^30^ It estimates the probability of an event (eg, appointment non-adherence) by fitting data to a logistic curve. Logistic regression assumes linear relationships between independent variables and the log odds of the dependent variable unless the model explicitly accounts for non-linearity.^31^^,^^32^

Decision Trees are machine learning algorithms generating inherently interpretable models. The algorithm works by recursively splitting the data into subsets based on the value of input features, creating a tree-like model of decisions or outcomes. This structure allows for easy interpretation and visualization of the decision-making paths, which are valuable for understanding how decisions or outcomes are reached and predicting them. The recursive split of the data is done such that it results in the highest information gain or the greatest reduction in impurity (ie, splitting the data into homogeneous categories).^33^

XGBoost is an advanced machine-learning algorithm that addresses complex data patterns through an ensemble model approach. It employs gradient boosting to sequentially construct a series of decision trees, each aiming to correct the errors of its predecessors.^34^ This technique incrementally combines predictions from multiple trees to enhance prediction accuracy. XGBoost has demonstrated superior performance in various healthcare and non-healthcare problems.^35–37^

Three models were trained and tested on the same dataset, allowing for a comparative analysis of their performance. We report performance measure values from the testing data. This approach evaluates not only their predictive accuracy but also their ability to generalize findings to new, unseen data, which is crucial for practical applications in healthcare settings. We also explored how the 3 methods differently utilize the key variables by representing the importance (or significance) of the variables incorporated into the models. We utilized the R version 4.3.2 to conduct our modeling and analysis.

### Performance measures

We evaluated model performance by employing multiple performance measures, including accuracy, sensitivity, precision, specificity, and the area under the curve (AUC). Accuracy measures the proportion of true results (both true positives and true negatives) among the total number of tested observations.^38^ However, accuracy does not distinguish between prediction error types, be they false positives or false negatives. Sensitivity evaluates the model’s ability to correctly identify true positives. Specificity assesses the model’s effectiveness in identifying true negatives. Precision measures the accuracy of positive predictions (ie, true positives among all positives). Lastly, AUC reflects the trade-off between sensitivity and specificity by plotting the false positive rate (1—specificity) against the true positive rate (sensitivity) at various prediction probability thresholds. An AUC value above 0.7 is considered acceptable for many applications, indicating that the model has a good measure of separability, while a value above 0.8 is deemed excellent.^29^ These measures are critical in healthcare settings, where the balance between correctly identifying conditions and minimizing false alarms can have significant implications for patient care and resource allocation.

### Feature importance

Understanding the importance of features in predictive modeling is crucial for interpreting model outputs and making informed decisions. In generalized linear models, such as logistic regression, the significance of a variable is often judged by the p-value of its coefficient. This provides an indicator of whether a variable significantly influences the model outcome.

In contrast, feature importance in decision trees is computed based on the reduction in impurity that each variable contributes to the splits it participates in across the tree. Unlike the Gini impurity ranging from 0 (perfect purity) to 0.5 (maximum impurity in a binary classification), Gini importance values do not have a fixed range while higher values indicate a greater importance or contribution of a variable to the tree construction process.

Lastly, XGBoost employs more sophisticated metrics to evaluate feature importance. The “Gain” of a feature indicates the improvement in model performance it brings, measuring a feature’s contribution to each node across all trees, weighted by the number of observations passing through those nodes.^34^ The “Cover” metric reflects the average number of observations affected by a feature across all trees where it appears, indicating the breadth of a feature’s impact.^33^ Meanwhile, “Frequency” describes how often a feature is used to split the data across all boosting rounds,^33^ with a higher frequency suggesting a feature’s recurrent utility in making splits. These metrics collectively offer insight into a feature’s influence within the XGBoost model prediction process. While Gain is often emphasized for its direct link to model accuracy enhancements, Cover and Frequency provide valuable context for comprehensive model interpretation and understanding feature interactions.

## Results

### Descriptive analysis results

**Figure 1.**
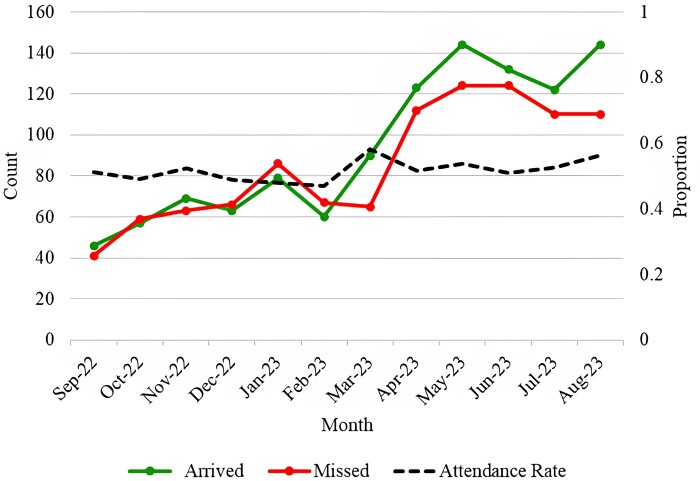
Monthly trend of post-discharge clinic (PDC) appointment status.

As Figure 1 shows, despite an increase in overall appointment volume, the PDC appointment attendance rate remained relatively constant at around the 50% level. This observation underscores the critical need for an effective appointment adherence strategy to address the persistently high rate of missed appointments.

Table 1 presents the univariate and bivariate analysis results, showing the distribution of demographic, socio-economic, and clinical variables across binary appointment status groups (arrived vs missed). The average age of the 2155 patients was 48.2 years (SD = 16.5). The majority of these patients were Black (52.6%), followed by White (39.5%), and Hispanic (3.9%). Most patients referred were female (53.9%), single (52.3%), and either covered by commercial insurance (47.7%) or uninsured/self-pay (18.8%).

**Table 1.** PDC patient characteristics.

	All	Arrived	Cancelled/no-show	*t*-test/Chi-square
**Demographic and socioeconomic factors**				
Age, mean (SD)	48.2 (16.5)	48.9 (16.4)	47.5 (16.6)	***t*** = **3.9**, ***P*** < **.05**
Race/ethnicity, *N* (% of total)				*χ* ^2^ = 10.4, *P* = .11
Black	1133 (52.6)	587 (52.0)	546 (53.2)	
White	851 (39.5)	455 (40.3)	396 (38.6)	
Hispanic	83 (3.9)	49 (4.3)	34 (3.3)	
Asian	39 (1.8)	18 (1.6)	21 (2.0)	
American Indian and Alaska Native	7 (0.3)	2 (0.2)	5 (0.5)	
Unknown	42 (1.9)	18 (1.6)	24 (2.3)	
Gender, *N* (% of total)				*χ* ^2^ = 0.2, *P* = .62
Female	1161 (53.9)	602 (53.3)	559 (54.5)	
Male	994 (46.1)	527 (46.7)	467 (45.5)	
Marital status, *N* (% of total)				*χ* ^2^ = 9.7, *P* = .14
Single	1128 (52.3)	588 (52.1)	540 (52.6)	
Married/life partner	645 (29.9)	363 (32.2)	282 (27.5)	
Divorced/separated	227 (10.5)	103 (9.1)	124 (12.1)	
Widowed	103 (4.8)	51 (4.5)	52 (5.1)	
Unknown	52 (2.4)	24 (2.1)	28 (2.7)	
Preferred language				***χ* ^2^** = **11.7**, ***P*** < **.05**
English	2052 (95.2)	1077 (95.4)	975 (95.0)	
Spanish	50 (2.3)	33 (2.9)	17 (1.7)	
Other or unknown	53 (2.5)	19 (1.7)	34 (3.3)	
Payer type				***χ* ^2^** = **20.8**, ***P*** < **.001**
Commercial	1028 (47.7)	585 (51.8)	443 (43.2)	
Medicare	336 (15.6)	172 (15.2)	164 (16.0)	
Medicaid	345 (16.0)	150 (13.3)	195 (19.0)	
Uninsured/self-pay	405 (18.8)	201 (17.8)	204 (19.9)	
Unknown	41 (1.9)	21 (1.9)	20 (1.9)	
Specific needs description				*χ* ^2^ = 7.9, *P* = .25
Not applicable	2103 (97.6)	1100 (97.4)	1003 (97.8)	
Wheelchair	9 (0.4)	2 (0.2)	7 (0.7)	
Other	43 (2.0)	27 (2.4)	16 (1.5)	
**Medical history**				
Diabetes	622 (28.9)	319 (28.3)	303 (29.5)	*χ* ^2^ = 1.3, *P* = .52
Myocardial infarction	264 (12.3)	123 (10.9)	141 (13.7)	*χ* ^2^ = 4.9, *P* = .08
Coronary artery disease	367 (17.0)	189 (16.7)	178 (17.3)	*χ* ^2^ = 1.0, *P* = .59
Chronic kidney disease	298 (13.8)	141 (12.5)	157 (15.3)	*χ* ^2^ = 4.4 *P* = .11
Hypertension	972 (45.1)	544 (48.2)	428 (41.7)	***χ* ^2^** = **10.1**, ***P*** < **.01**
Chronic obstructive pulmonary disease (COPD)	259 (12.0)	121 (10.7)	138 (13.5)	*χ* ^2^ = 4.7, *P* = .10
Venous thromboembolism pulmonary embolism	238 (11.0)	107 (9.5)	131 (12.8)	***χ* ^2^** = **6.8**, ***P*** < **.05**
Asthma	357 (16.6)	169 (15.0)	188 (18.3)	*χ* ^2^ = 5.2, *P* = .07
Anxiety	660 (30.6)	316 (28.0)	344 (33.5)	***χ* ^2^** = **8.6**, ***P*** < **.05**
Autoimmune disorder	130 (6.0)	68 (6.0)	62 (6.0)	*χ* ^2^ = 0.9, *P* = .63
Depression	479 (22.2)	227 (20.1)	252 (24.6)	***χ* ^2^** = **7.0**, ***P*** < **.05**
Dyslipidemia	604 (28.0)	327 (29.0)	277 (27.0)	*χ* ^2^ = 2.0, *P* < .37
Heart failure	347 (16.1)	161 (14.3)	186 (18.1)	***χ* ^2^** = **6.8**, ***P*** < **.05**
**Appointment scheduling factor**				
Lag days, mean (SD)	6.6 (14.1)	4.5 (7.5)	8.8 (18.6)	***t*** = **52.5**, ***P*** < **.001**

Bold indicates significant correlation. Abbreviations: PDC, post discharge clinic.

The bivariate analysis (Table 1, columns 3-5) reveals significant differences in patient characteristics based on appointment status. For instance, the average age of patients with “arrived” appointments was significantly greater than those with missed appointments (*t* = 3.9, *P* < .05). Individuals using languages other than English and Spanish as their first language were more likely to miss appointments (*χ*^2^ = 11.7, *P* < .05), as were patients with Medicaid or uninsured/self-insured (*χ*^2^ = 20.8, *P* < .001).

Additional statistical differences were identified through chi-square analysis. Patients with arrived appointments had a higher prevalence of hypertension (48.2%) than those who missed their appointments (41.7%) (*χ*^2^ = 10.1, *P* < .01). Conversely, anxiety and depression were more prevalent among patients who missed their appointments, suggesting these conditions as potential barriers to accessing PDC services (*χ*^2^ = 8.6, *P* < .05, *χ*^2^ = 7.0, *P* < .05, respectively). Lastly, the average number of days between appointment scheduling and occurrence (lag days) was 6.6 days. A longer interval was significantly associated with a higher likelihood of missing appointments (*t* = 52.5, *P* < .001).

### Prediction outcomes

**Figure 2.**
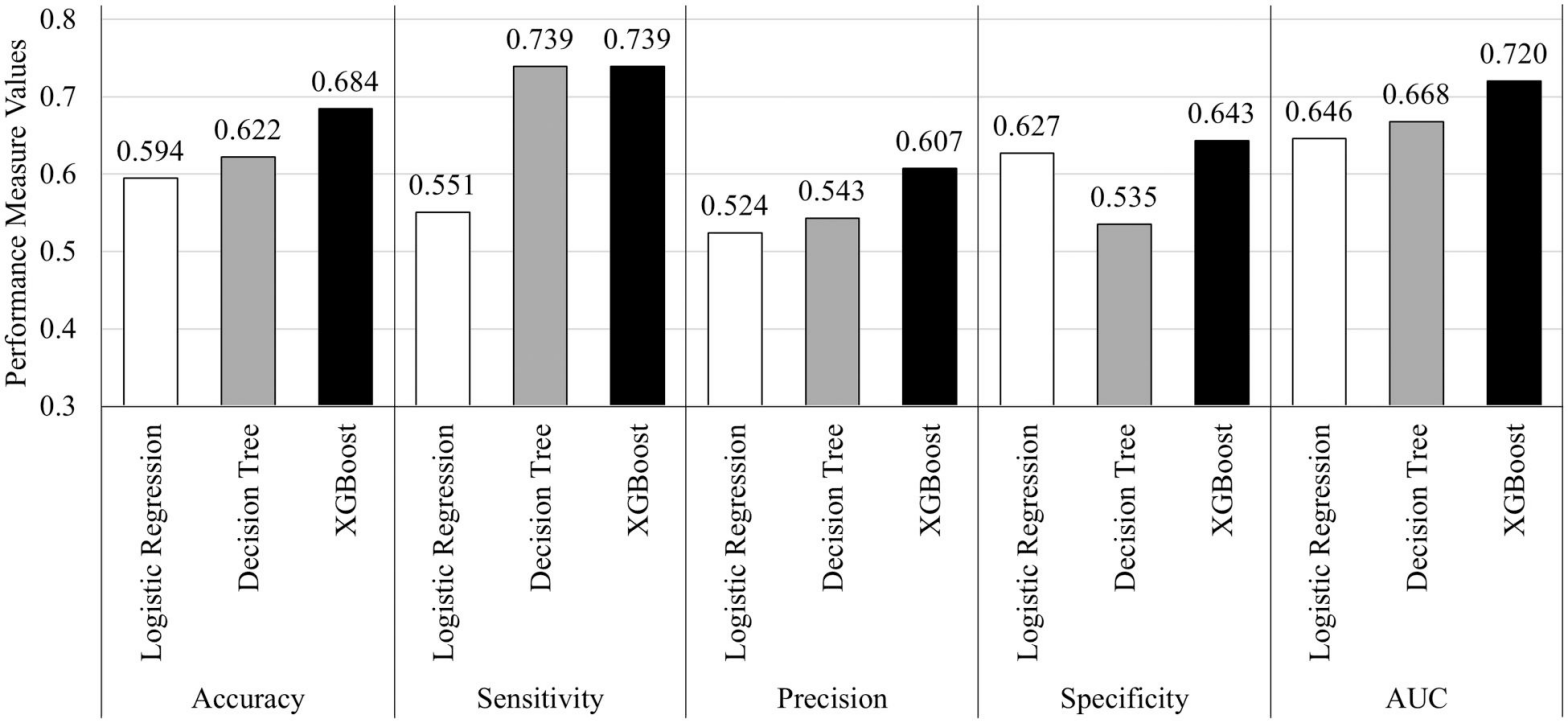
Model performance comparison.

As illustrated in Figure 2, the XGBoost model demonstrated superior performance in identifying cases prone to missed appointments. XGBoost demonstrated higher accuracy (68.4%), specificity (64.3%), precision (60.7%), and AUC (72.0%) than the other 2 models. The decision tree and XGBoost were equally as sensitive with 73.9% each. Nevertheless, XGBoost’s AUC value, serving as a summary measure of the model’s ability to balance sensitivity and specificity, indicates a stronger overall discriminative power in distinguishing between patients likely to attend or miss appointments. This underscores XGBoost’s potential impact on appointment adherence in a new clinic venture.

### Logistic regression variable odds ratio

Table 2 reports logistic regression results. Demographic and socio-economic variables were significantly associated with appointment status. Divorced/separated and widowed individuals were more likely to miss appointments [odds ratio (OR) 1.57 (*P* < .05) and 1.76 (*P* < .05), respectively]. Additionally, payer type emerged as a critical factor, with Medicaid (OR 1.67, *P* < .001) and uninsured/self-paying (OR 1.44, *P* < .05) persons having higher odds of non-attendance. In contrast, hypertension was significantly associated with lower odds of missing appointments (OR 0.58, *P* < .001), indicating that specific health conditions might differentially influence appointment adherence. The number of days between appointment scheduling and occurrence (lag days) remained highly significant (*P* < .001) after controlling for all other factors in the model, underscoring its impact on appointment adherence.

**Table 2.** Summary of logistic regression results.

Variable	Odds ratio	95% CI	*P*-value
Intercept	8.7e-08	(0-10.0)	.99
**Demographic and socioeconomic factors**			
Age	0.99	(0.98-1.01)	.41
Race/ethnicity			
Black	Reference		
White	1.03	(0.82-1.30)	.80
Hispanic	0.63	(0.21-1.81)	.39
Asian	1.22	(0.58-2.56)	.60
Unknown	4.21	(0.93-29.81)	.09
Gender			
Female	Reference		
Male	1.01	(0.81-1.25)	.92
Marital status			
Married/life partner	Reference		
Single	1.09	(0.85-1.41)	.50
Divorced/separated	1.57*	(1.06-2.33)	<**.05**
Widowed	1.76*	(1.04-2.97)	<**.05**
Unknown	1.02	(0.47-2.18)	0.96
Preferred language			
English	Reference		
Spanish	0.44	(0.21-1.44)	.23
Other	1.45	(0.88-2.36)	.32
Payer type			
Commercial	Reference		
Medicare	1.31	(0.94-1.84)	.11
Medicaid	1.67***	(1.23-2.25)	<**.001**
Uninsured/self-pay	1.44*	(1.09-1.91)	<**.05**
Specific needs description			
Not applicable	Reference		
Wheelchair	8.96^†^	(1.10-48.0)	.08
Other	1.61	(0.43-6.94)	.49
**Medical history**			
Diabetes	0.90	(0.34-2.34)	.83
Myocardial infarction	1.12	(0.77-1.63)	.55
Coronary artery disease	0.90	(0.63-1.26)	.53
Chronic kidney disease	1.44†	(0.98-2.11)	.06
Hypertension	0.58***	(0.45-0.74)	<**.001**
COPD	1.22	(0.86-1.73)	.27
Venous thromboembolism pulmonary embolism	1.63	(0.83-3.22)	.16
Asthma	1.12	(0.84-1.50)	.42
Anxiety	1.20	(0.92-1.57)	.17
Autoimmune disorder	0.95	(0.61-1.45)	.80
Depression	0.86	(0.64-1.17)	.34
Dyslipidemia	1.09	(0.82-1.44)	.57
Heart failure	1.30	(0.91-1.86)	.14
**Appointment scheduling factor**			
Lag days	1.05***	(1.03-1.07)	<**.001**

Bold indicates significant correlation. Significance codes: “***” .001; “**” .01; “*” .05; “†” .1

### Decision tree model feature importance

**Figure 3.**
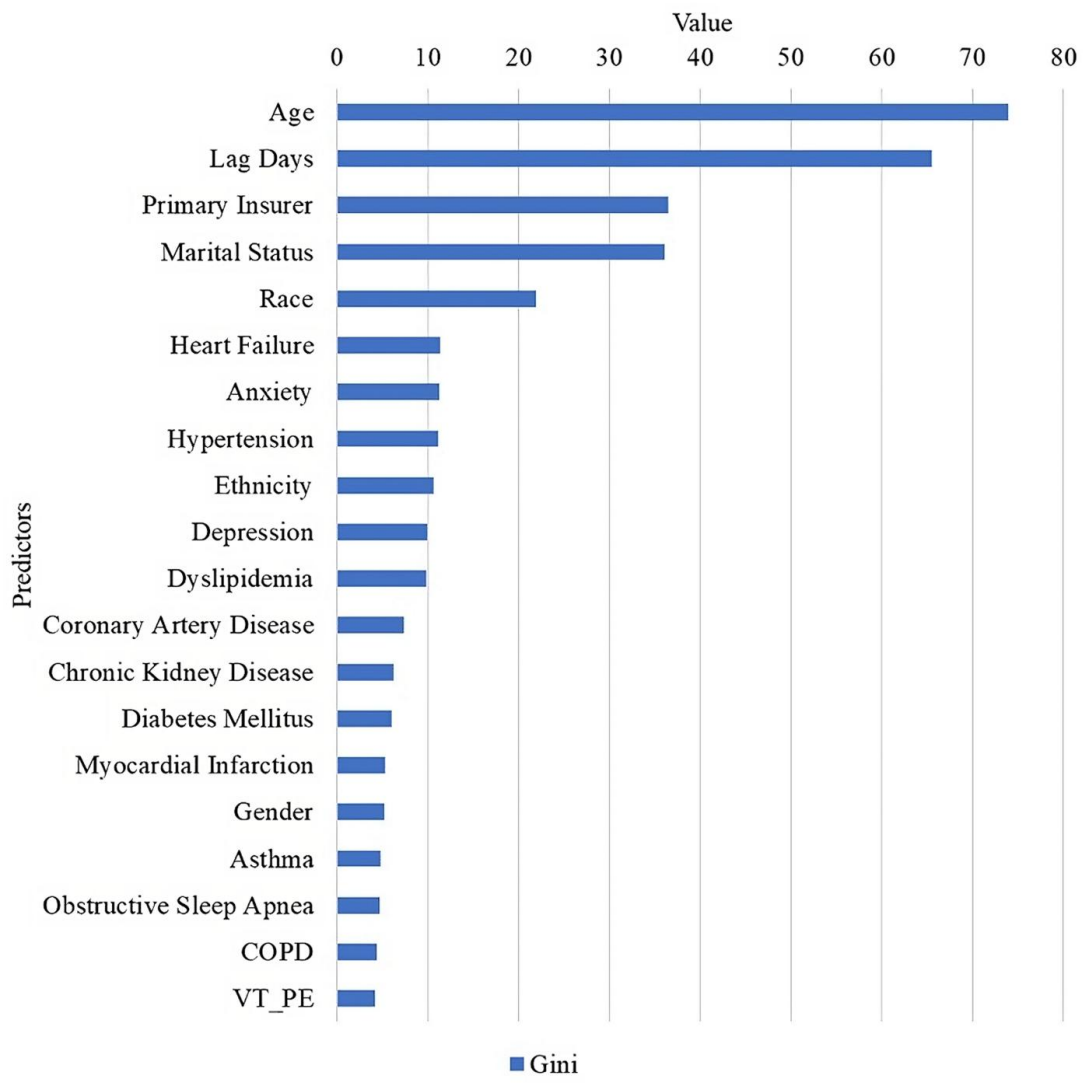
Gini feature importance from the decision tree model.

Figure 3 presents the Gini importance values for the top 20 variables as identified by the decision tree method. This list encompasses a mix of scheduling-related, demographic, socio-economic, and clinical characteristics. Distinct from the findings in logistic regression analysis, age appeared to be highly important in the decision tree model. This indicates that age may exhibit a non-linear effect or interact with other variables. It is important to note that a higher feature importance does not directly suggest a variable contributes to missed appointments, unlike the OR from logistic regression, which indicates the direction of association. Instead, it signifies the variable’s capacity to distinguish between the arrived and missed appointment classes. For instance, although hypertension emerged as a significant feature, it may indicate a higher likelihood of attendance among individuals with hypertension, thus aiding in identifying likely attendees in the binary classification.

**Figure 4.**
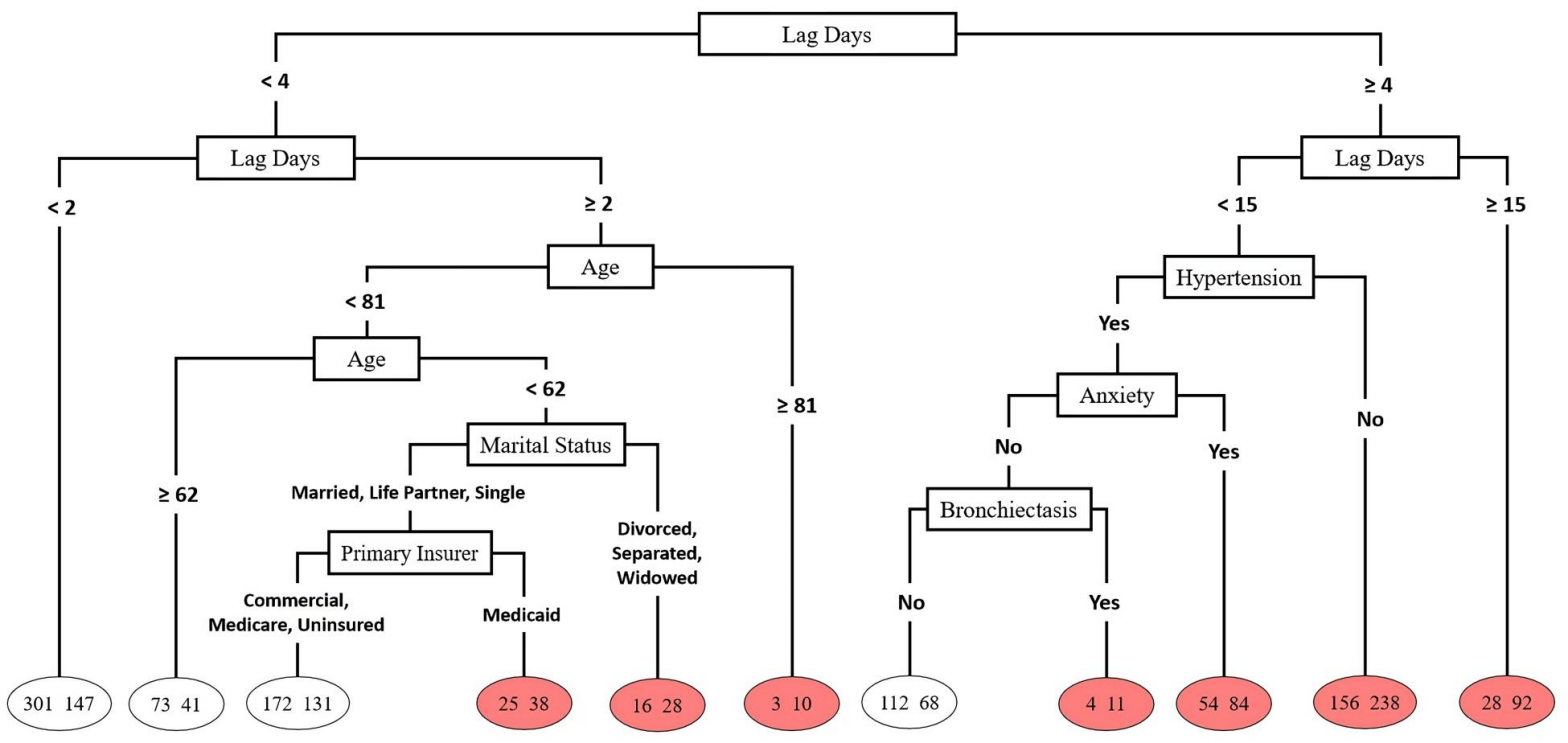
Decision tree structure.

Figure 4 visualizes the decision tree structure created by our predictive decision tree model. Lag days and age appear to be the most influential variables. Demographic and socioeconomic variables are associated with appointment adherence among patients with more than 2 but less than 4 lag days, but medical history variables are associated with adherence among those with more than 4 but less than 15 lag days.

### XGBoost model feature importance

**Figure 5.**
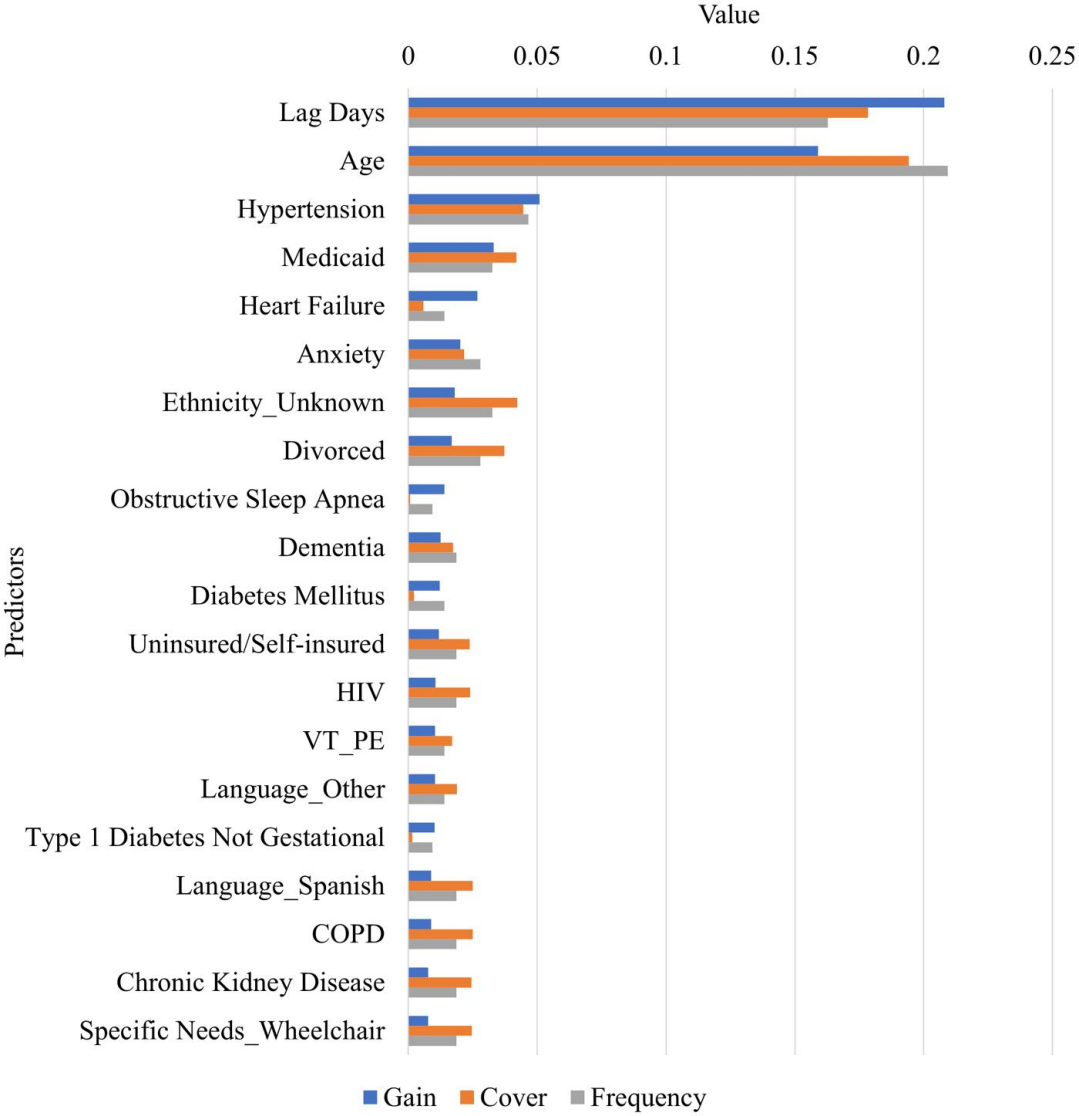
Feature importance from the eXtreme Gradient Boosting (XGBoost) model.

Figure 5 presents the top 20 variables in terms of importance, as identified by the XGBoost method. Similar to the decision tree model, the list encompasses a mix of variable types. Lag days and age were identified as the most informative, followed by insurance status, marital status, and preferred language, highlighting the influence of socio-economic factors.

### Sensitivity analysis on probability threshold values

**Figure 6.**
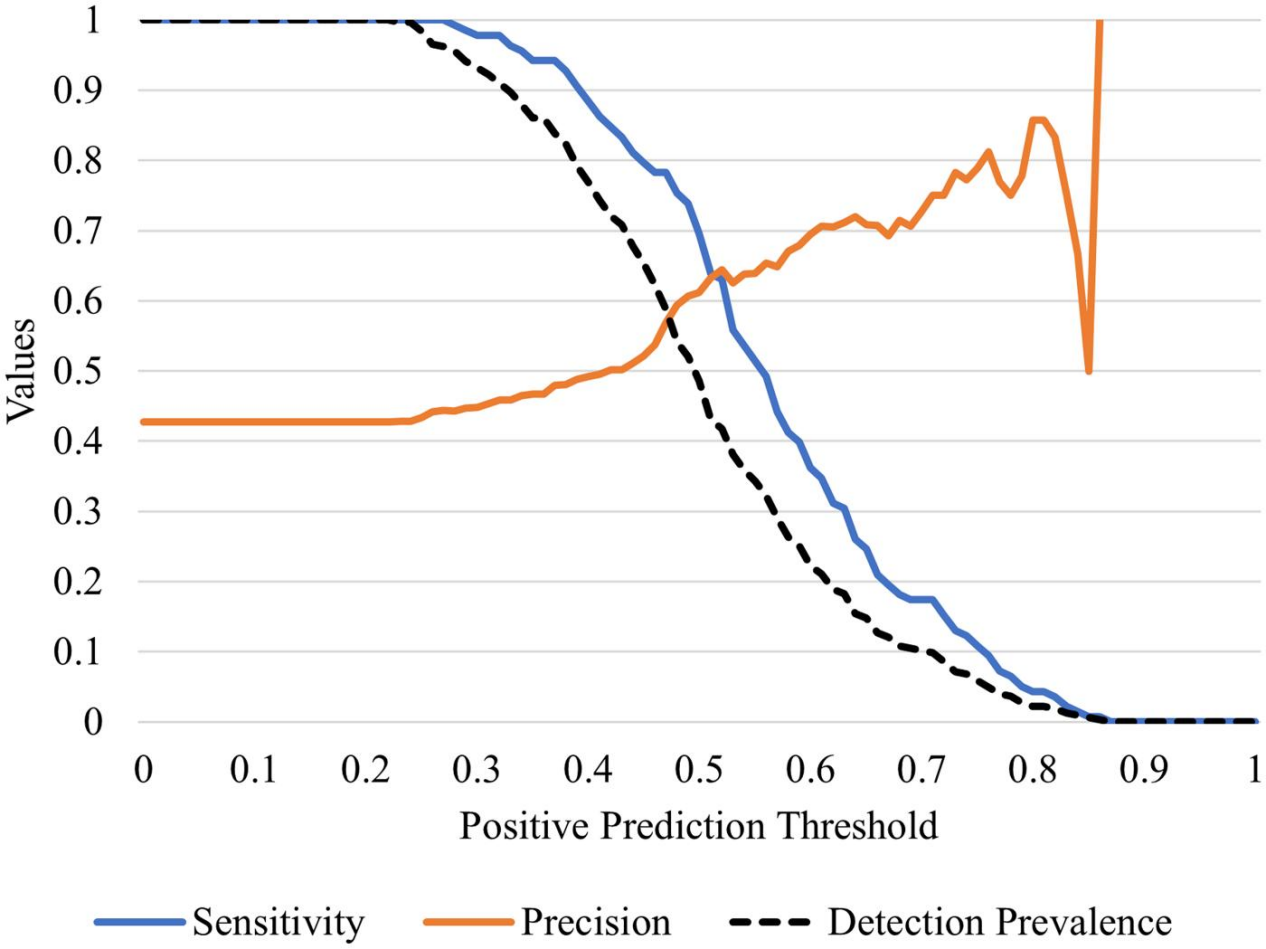
Trade-off between sensitivity, detection prevalence, and precision.

Figure 6 represents that as the probability threshold (ie, the probability at which an appointment is predicted to be missed) increases, the model predicts fewer patients to miss their appointments (resulting in lower detection prevalence), leading to a decrease in sensitivity but an increase in precision. For example, setting the positive prediction threshold at 0.4 allows our model to detect 88.4% of positive cases with a precision of 49.0%. On the other hand, with the threshold set at 0.6, the model’s sensitivity drops to 34.8% while its precision increases to 70.6%.

As described, a lower threshold increases sensitivity but may include more false positives, while a higher threshold enhances precision but may miss identifying individuals at risk. Understanding this trade-off is crucial for optimizing strategies to improve patient engagement. Reflecting on this analysis enables healthcare providers to make informed decisions that align with their operational goals and patient care standards in the context of fiscal capacity.

## Discussion

In this study, we applied a data-driven machine learning method to identify patients who are more likely to miss a scheduled appointment at a new PDC and compared this approach to more traditional prediction methods. XGBoost stands out for its ability to handle non-linear relationships and intricate interactions between variables,^39^^,^^40^ which are common in health-related data.^41^^,^^42^ This approach allows for capturing a broader spectrum of data patterns and interactions, providing more nuanced results and potentially leading to more accurate and reliable predictions compared to traditional logistic regression, as indicated by superior performance on all model performance measures compared to logistic regression and decision tree modeling. Leveraging the predictive capabilities of XGBoost, our findings underscore the potential of machine learning to enhance clinic operations by better identifying those at higher risk of missing future appointments. This approach allows for new opportunities for prioritizing patients for targeted interventions to improve healthcare operations and ultimately patient outcomes.

Our findings shed light on the significant role that lag time, age, insurance status, marital status, and medical history have in predicting appointment adherence. For instance, despite the PDC offering financial assistance to uninsured patients, disadvantageous insurance statuses (such as Medicaid and uninsured) still posed barriers to accessing PDC appointments. Because these factors may impact patient engagement beyond appointment attendance (eg, cultural competence of the healthcare system and financial literacy), our findings could be valuable for developing additional patient engagement strategies and may be applicable across other clinic settings. From the modeling perspective, distinct from the findings in logistic regression analysis, the results from the XGBoost and decision tree models consistently identified age as a highly important variable. The consistency between the decision tree and XGBoost model results suggests the robustness of the results. The significance of the age variable identified by both the decision tree and XGBoost method testifies to the models’ advanced capacity for detecting intricate patterns. For healthcare clinical and operational leaders, these findings highlight the importance of minimizing the lag time and particular attention to older (≥81) or younger (<62) patients who, as groups, were more likely to miss PDC appointments in our study.

Our analysis also revealed the necessity of optimizing the sensitivity and precision trade-off in predictive models to enhance the effectiveness of appointment adherence interventions. A clinic can adjust the prediction threshold based on their financial resources and physical and human resource capacity to follow up on predicted no-shows or their prioritization of minimizing missed appointments versus avoiding unnecessary follow-ups. This decision impacts resource allocation, the effectiveness of patient engagement efforts, and ultimately, the clinic’s operational efficiency and patient care quality. This optimization ensures the detection of patients at increased risk of missing appointments while minimizing false positives that could lead to resource misallocation and alarm fatigue among healthcare professionals.^43^^,^^44^ Fine-tuning the prediction threshold is paramount for deploying efficient and sustainable patient engagement strategies, ensuring that patient engagement interventions are both targeted and impactful. The optimization of a prediction threshold has also been discussed in the ED admission prediction setting.^45^

The dynamic nature of prediction thresholds demands continuous evaluation and adjustment of model parameters based on patient outcomes and new data. This adaptability is vital in healthcare practices. Integrating advanced predictive analytics into clinical and operational workflows marks a pivotal shift toward more proactive and personalized healthcare. However, realizing these benefits requires addressing potential optimization problems with these tools and enhancing their usability in partnership with healthcare professionals.

Our findings have important implications for both post-discharge care operations as well as strategies to improve patient engagement such as appointment adherence. By demonstrating the value of machine learning models like XGBoost in predicting appointment adherence, our study contributes to the growing field of data-driven healthcare decision-making. Strategies such as these hold promise for allocating resources more judiciously, tailoring interventions to individual patient profiles, and ultimately elevating the quality and efficiency of care.

### Limitations

This study is subject to several limitations. First, the study findings are based on EHR data from a single institution, potentially limiting their applicability to broader post-discharge care settings. However, we note that the variables included in prediction models are ubiquitous in healthcare settings. Second, the study’s sample size was relatively limited for predictive analytics research, a reflection of our intentional focus on supporting patient engagement for a new clinic venture, where patient engagement mechanisms were nascent and system-wide awareness was limited. This prompts consideration of how our findings may not be generalizable for more mature clinics with established patient engagement protocols and system-wide visibility, though the modeling approaches employed can easily be applied in other settings and compared to our findings. Third, logistic regression, decision trees, and XGBoost were chosen as representatives of traditional statistical methods and advanced machine learning techniques, respectively. Yet, other modeling approaches might provide additional insights into predictive accuracy or interpretability. Despite these limitations, our study offers valuable insights for healthcare institutions aiming to improve post-discharge appointment adherence.

## Conclusion and future work

This study has demonstrated the potential for predictive analytics to prioritize patients for enhanced engagement initiatives in transitional ambulatory care by identifying patients at increased risk of missing appointments. Highlighting key variables that influence appointment adherence, our findings offer actionable insights for developing focused interventions delivered to prioritized patients to improve post-discharge care. To the best of our knowledge, this work is among the first to apply machine learning to inform interventions that address post-discharge appointment adherence in new clinical ventures. An important next step involves integrating these models with intervention feasibility studies (eg, pilot implementation) to evaluate their potential impact on patient outcomes, moving beyond prediction to enhancing patient engagement and care continuity. By advancing predictive analytics in healthcare, our work sets the stage for more personalized and effective post-discharge interventions, ultimately contributing to improved healthcare delivery and patient well-being.

## Data Availability

The EHR datasets analyzed during the current study are not publicly available because they are governed by HIPAA regulations at the University of Alabama at Birmingham.
